# Teen Actors Teaching Communication Skills

**DOI:** 10.7759/cureus.19515

**Published:** 2021-11-12

**Authors:** Joni A Hemond, Kathleen M Franchek-Roa, Deirdre A Caplin, Wendy L Hobson

**Affiliations:** 1 Department of Pediatrics, University of Utah, Salt Lake City, USA

**Keywords:** standardized patients, medical education, provider-patient communication, communication, adolescent medicine

## Abstract

Introduction

To provide high-quality care, physicians must effectively communicate with adolescents while addressing difficult and sensitive subjects. Our program aimed to (1) cost-effectively incorporate teenage actors into a pediatric simulation program and (2) increase residents' self-perceived comfort level with adolescent patients by practicing interview skills with teens.

Methods

In 2013, the authors established a Teen Acting Program, in which volunteer theater students created and simulated patient characters and provided feedback to learners. Residents on the adolescent medicine rotation participated in the program, completed a survey on self-perceived comfort level with adolescent patients, and answered open-ended questions regarding the program.

Results

A total of 70 residents participated in the program and pre-survey; 46 completed both the pre- and the post-survey. Of 46 participants, 37 (80%) reported that the program was helpful and four (9%) stated it was the best thing about the rotation; 35 (80%) described the program as “high-yield” for the time spent. Between the pre- and post-surveys, residents demonstrated statistically significant improvement in comfort interacting with adolescent patients, addressing confidentiality, and taking histories on sexuality, substance use, mental health, diet, and safety, whether they were the interviewer or observer. Residents that reported the teens taught them specific skills and concepts related to communication. A calculation of program costs demonstrated a 10-fold decrease in cost from traditional simulation patient encounters.

Conclusion

The Teen Acting Program was cost-effective and improved resident comfort with interviewing adolescents about sensitive topics, while giving adolescent actors experience honing their acting skills.

## Introduction

Adolescent health visits are uniquely challenging. Physicians in training can struggle with asking about mental health, eating disorders, sexual health, and peer influence topics [1]. To communicate effectively with adolescents, trainees must maintain adolescent-centered care while addressing conditional confidentiality, risk, and resilience [2,3]. On busy clinical services, educating residents in this type of communication can be difficult; therefore, simulated patients may be useful for skills training.

Studies using simulated adolescent patients have shown improvements in objective and self-perceived knowledge, comfort level with interviewing, and clinical skills [4-9]. Learners receiving structured feedback further improved long-term interview skills over those who did not receive feedback [5,10]. Previous programs were not part of an ongoing adolescent curriculum, not with residents, and unlikely to be sustainable due to cost. Our aims were to (1) cost-effectively incorporate teenage actors into a simulation program for pediatric residents, allowing for feedback from the teenagers, and (2) increase residents' self-perceived comfort level with adolescent patients by practicing interview skills with high school actors.

## Materials and methods

In 2013, we established the Teen Acting Program to train theater students from a local high school to create patient characters, act in these roles, and give feedback to residents.

Actors

Collaborating with the high-school administration, we introduce the program at the beginning of the school year. Acting teachers recommend students, and we select four to six students annually. The student actors volunteered to participate but did not receive payment nor course credit, yet they did put the experience on their resumes and applications and often asked the faculty for a letter of recommendation.

High-school actor training sessions

Students receive in-person training with follow-up work at home (Table 1). Students select from a list of possible topics and create the case based on personal experience, research, and discussion with the group. They pilot the case with residents and the faculty supervisor and make changes based on suggestions. A pediatrician mentor provides the teens with coaching about medical components. Cases have included sexual promiscuity, LGBTQ identity, substance use, mental health issues, social media, and eating disorders. Cases change annually as the teen actors change.

**Table 1:** Educational training sessions HEADSSS: Home, Education/Employment, Activities, Drugs, Sex/Relationships, Self harm/Depression, Safety/Abuse

Teen actor training	Content
Introduction	Importance. HEADSSS assessment [11]. Emphasize that cases should be completely fictional. Create a list of potential cases.
Feedback	Examples of good and poor feedback. Emphasize use of specific examples. Highlight verbal and non-verbal communication.
Create cases	Create details using HEADSSS as a framework. Some work done outside of sessions.
Cases with faculty leader	Review case. Ensure that it is realistic and appropriate. Make changes accordingly.
Practice cases	Practice being the “patient” and the “doctor”. Continue to refine details and make changes.
Resident training	Content
Introduction	Introduce high-school students to residents. Faculty reviews HEADSSS assessment with residents. Faculty explains the program and what residents should get out of it.
Interview	One resident conducts an interview, while others in attendance observe. High-school students not in character take notes. Faculty should interrupt if either the actor or the resident is having anxiety or difficulty.
Feedback	High-school students give feedback on the interview with the resident. Faculty gives feedback to resident.
Debrief/reflection	Residents ask the students questions. Students are dismissed and residents discuss the experience with the faculty leader.

Resident educational sessions

Each session is comprised of two to three residents on their adolescent medicine rotation, a pediatric faculty mentor, and between four and six teen actors (Table 1). We hold 60- to 90-minute sessions in a high-school classroom or in a clinic. Sessions include one to two interviews; residents take turns interviewing and observing. The teen actor gives the resident direct verbal feedback on body language, phrasing, and appropriateness of questions. The other teens and the mentor use a structured form for additional observations and feedback. The mentor debriefs and reflects with actors, observers, and residents at the end of each session.

Evaluation design

The University of Utah Institutional Review Board exempted this study (UUIRB_00065432). We hypothesized that the program would increase residents’ perceived comfort interacting with adolescent patients, addressing confidentiality, and taking histories on sexual activity, substance use, mental health, diet (including eating disorder behaviors), social media, and safety (including dating violence). De novo, we created an 11-question pre-survey and a nine-question post-survey to evaluate the program. For the comfort-level questions, a five-point Likert scale was used, ranging from very uncomfortable to very comfortable. Faculty pilot tested the survey prior to distribution to the learners. No changes in the survey were made during the study period.

Pediatric residents consented to the study; they answered surveys on the first (pre-survey) day and last (post-survey) day of the adolescent rotation. Both surveys asked about comfort level with the targeted topics. The post-survey included questions about resident experience with the program, which patients were interviewed/observed by each resident, and the program's usefulness. The post-survey included a final open-ended question: “What specific phrases and/or strategies did you learn work well in interacting with teenagers?” We used McNemar’s test to compare pre- and post-test responses. In an adjunct analysis, we summarized open-ended comments separately to describe important lessons learned [11]. All authors reviewed the comments, created concept categories, and came to consensus.

## Results

Since 2013, pediatric and family medicine residents have interviewed volunteer teen actors as part of the program; during these eight years, the program has been sustainable.

During the 29-month evaluation period, 71 residents participated in the program and 70/71 (99%) completed the pre-survey. Of the 71 residents, 46 (65%) completed both the pre- and post-survey questions, of whom 41 (89%) were categorical pediatric residents and five (11%) were combined residents. Thirty-seven (80%) of 46 reported that the program was helpful and four (9%) stated that it was the best thing about the rotation; 35 (80%) described the program as “high-yield” for the time spent.

**Figure 1:**
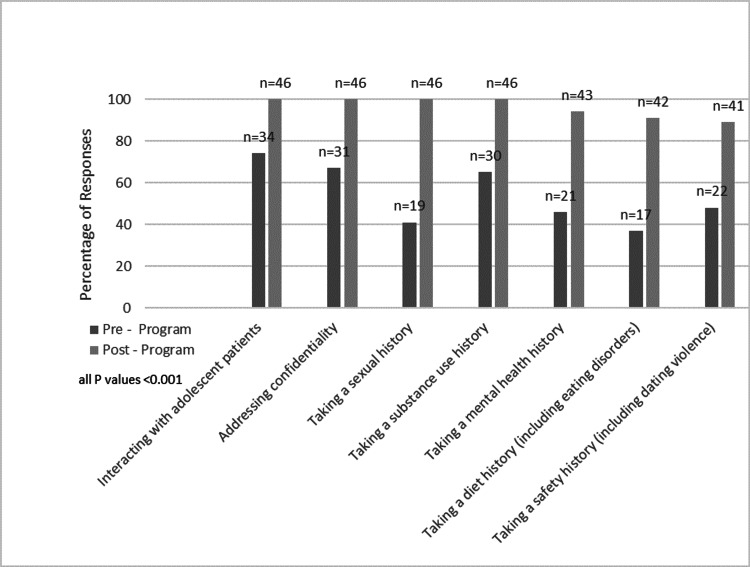
Residents reporting comfortable/very comfortable with skill

Between the pre- and post-surveys, residents demonstrated statistically significant improvement in comfort discussing all topics, whether they were the interviewer or observer. Residents interviewing teen characters perceived improvements discussing mental health (86%; 18/21), substance use (94%; 17/18), sexual behavior (90%; 18/20), disordered eating (69%; 9/13), and safety (100%; 15/15). Resident observers reported perceived improvements discussing mental health (100%; 6/6), substance use (100%; 14/14), sexual behavior (94%; 16/17), disordered eating/diet (80%; 4/5), and safety (100%; 10/10) (Figure 1).

Overall, 95% (44/46) of residents reported that the teen actors’ feedback was helpful. Open-ended responses suggested that residents wanted more interaction with the teens and learned important skills related to body language and confidentiality statements. Residents reported the teens taught them specific skills and concepts related to communication (Table 2). The concepts gleaned from the open-ended question corroborated the survey findings.

**Table 2:** Learning concepts from the question: what specific phrases and/or strategies did you learn work well in interacting with teenagers?

Concept	Representative Answer
Avoid medical jargon	Don't sound too “doctorly.” To say “boss” instead of attending.
Use clear, direct language	Saying “sex” instead of sexual intercourse or other awkward phrases. Just be upfront and ask the question you want/need to ask. I learned critical phrasing to avoid, to be direct, and to avoid making premature summarizing statements that may come across as judgmental.
Pace interview naturally	Always start out with "what do you like to do for fun?" don't just go down the questionnaire but work everything in gradually. Establish rapport by talking with them about what they are passionate about. Just asking questions rather than pre-phrasing “we are going to talk about some private issues now…”
Address adolescent-specific issues	Making sure kids put their phones away. To ask who the adolescent brought with them instead of assuming it’s a parent. Understand their concrete thinking tendency and employ more motivational interviewing than telling. Open-ended questions seem even more valuable for teens. Asking about their sexual orientation and not assuming based on if they are dating someone now. Making sure to always ask about the age of their partners and how they developed these relationships. My posture can drastically change how the adolescent feels during the interview.
Include new phrases learned	Would you say you’re happy most of the time or sad, or somewhere in between? How often do you cry in the average week? What’s the worst/best part of school? Have you dated anyone yet? “My other patients who…” helps teens feel that their problem is not unique. Specifically, I do not ask about “drugs and alcohol” but rather split the two up and ask specific questions. To ask about prescription medications prior to illicit drug use was a good lead-in.

Anecdotally, teen actor volunteers reported their roles as simulated patients are of utmost importance, as they teach doctors who are often uncomfortable with sensitive topics. The teen actors noted that they benefited from the program by practicing “long-form improvisation” acting skills, developing characters, learning about adolescent issues, watching residents’ skills improve, and being exposed to the medical field.

Feasibility

Space at the high school or clinic and the teen actor time is donated in-kind. Faculty time above a traditional simulation program was four hours a year (approximately $400) to work with the teen actors. The adolescent rotation administrative coordinator scheduled the sessions as part of her existing responsibilities. Breakfast costs approximately $300 per year. Total cost was approximately $700. In contrast, the estimated project costs using our clinical skills teaching center would be $7,000 per year (standardized patients are paid two hours minimum for $36/hour, plus “case study” time, and the clinical skills center charges $72/hour) [12].

## Discussion

Through a novel Teen Acting Program, interviewing and observing simulated teen patients, pediatric residents reported improving their communication skills and comfort level with adolescent health issues. Because residents often feel ill equipped to interact with teen patients [1,2], we designed an educational intervention to meet adolescent patient [3], program [13], and resident needs, and Accreditation Council for Graduate Medical Education (ACGME) [14] standards (particularly around communication). The residents reported increased comfort level around a variety of adolescent issues.

The format of the educational sessions allowed the residents to have time interacting with teenagers, who were trained to give constructive feedback. Previous literature had demonstrated that directed feedback from actors improved long-term interview skills [5,10], and we found not only increased comfort levels but also other unintended benefits arose during the Teen Acting Program. During debriefing sessions, residents queried the teenagers about unclear ethical situations and best approaches. Because of the teen feedback, pediatrician mentors have made alterations in clinical work and teaching. For example, the teenagers suggested modifications to a typical adolescent interview [15] to improve interview flow, which were incorporated into adolescent medical interviews templates at our institution. The concepts delineated from the open-ended answer can be used for future teaching, using direct words from adolescents.

With educational costs increasing and budgetary constraints, meeting standards [14] has become more difficult. The Teen Acting Program is cost-effective and sustainable. Much of the strength of the program lies in its cost structure. Using teen actors, who also benefitted from the experience, resulted in a cost that was 1/10th of a typical simulated patient scenario at our hospital. The teen actors were credible, creating a rich experience for residents. The teens reported feeling as though they have made a difference.

Limitations

Results are self-reported rather than observed behavior changes. The post-survey may not have been representative as the response rate was only 65%. The program was limited to one pediatric residency program, and teen acting resources may not be readily available for other programs. As the program is part of an adolescent rotation, changes in comfort may not be entirely attributable to the Teen Acting Program.

This study was limited in scope. Future study opportunities include observed encounters in vivo in a clinical setting and as an observed structured clinical examination to demonstrate changes in behavior. Using a single administration retrospective pre-post survey could improve the response rate while overcoming possible response shift bias [16]. The teen actor experiences could be explored formally. An analysis of the feedback forms from the teen actors and the faculty could provide a richer description of lessons learned.

## Conclusions

Using teen actors as simulated patients was a mutually beneficial arrangement for teaching and learning. The Teen Acting Program provides a unique setting to give feedback to learners and evaluate communication skills. Teen actors shared their expertise with medical professionals and hone their acting skills. Residents engaged with a teen trained to provide honest feedback that was directly applicable to clinical practice.

Since the program uses volunteer teen actors, its financial costs are low, it provides a relatively authentic experience, and it has reported benefits. Medical schools and residency programs can replicate the program to teach important skills with a limited budget.

## References

[1] Kershnar R, Hooper C, Gold M, Norwitz ER, Illuzzi JL (2009). Adolescent medicine: attitudes, training, and experience of pediatric, family medicine, and obstetric-gynecology residents. Yale J Biol Med.

[2] White B, Viner RM (2012). Improving communication with adolescents. Arch Dis Child Educ Pract Ed.

[3] Alderman EM, Breuner CC (2019). Unique needs of the adolescent. Pediatrics.

[4] Haist SA, Wilson JF, Lineberry MJ, Griffith CH (2007). A randomized controlled trial using insinuated standardized patients to assess residents' domestic violence skills following a two-hour workshop. Teach Learn Med.

[5] Macdonald M MJ, Mann K, Blake K (2014). Improving medical student's confidence regarding adolescent interviewing. Pediat Therapeut.

[6] Sanci LA, Coffey CM, Veit FC, Carr-Gregg M, Patton GC, Bowes G, Day N (2000). Effects of an educational intervention for general practitioners in adolescent health care principles: a randomized controlled study. West J Med.

[7] Feddock CA, Hoellein AR, Griffith CH, Wilson JF, Lineberry MJ, Haist SA (2009). Enhancing knowledge and clinical skills through an adolescent medicine workshop. Arch Pediatr Adolesc Med.

[8] Fallucco EM, Hanson MD, Glowinski AL (2010). Teaching pediatric residents to assess adolescent suicide risk with a standardized patient module. Pediatrics.

[9] Gamble A, Bearman M, Nestel D (2016). A systematic review: Children &amp; Adolescents as simulated patients in health professional education. Adv Simul (Lond).

[10] Bourget G, Joukhadar N, Manos S, Mann K, Hatchette J, Blake K (2018). Adolescent interviewing skills: effect of feedback. Clin Teach.

[11] LaDonna KA, Taylor T, Lingard L (2018). Why open-ended survey questions are unlikely to support rigorous qualitative insights. Acad Med.

[12] (2021). Room Rental Fees. https://ehseb.utah.edu/scheduling/room-rental-fees/..

[13] Zumwalt AC, Carter EE, Gell-Levey IM, Mulkey N, Streed C Jr, Siegel J (2021). A novel curriculum assessment tool, based on AAMC competencies, to improve medical education about sexual and gender minority populations [Online ahead of print]. Acad Med.

[14] Accreditation Council for Graduate Medical Education (2021). ACGME Program Requirements for Graduate Medical Education in Pediatrics. https://www.acgme.org/globalassets/pfassets/programrequirements/320_pediatrics_2021v2.pdf..

[15] Katzenellenbogen R (2005). HEADSS: the "review of systems" for adolescents. Virtual Mentor.

[16] Geldhof GJ, Warner DA, Finders JK, Thogmartin AA, Clark A, Longway KA (2018). Revisiting the utility of retrospective pre-post designs: The need for mixed-method pilot data. Eval Program Plann.

